# Perception, attitudes, and knowledge on infestation and management of bed bugs in major cities of Indonesia: A cross-sectional online survey

**DOI:** 10.1371/journal.pone.0288682

**Published:** 2023-07-27

**Authors:** Dita Meisyara, Ikhsan Guswenrivo, G. Veera Singham

**Affiliations:** 1 Centre for Chemical Biology, Universiti Sains Malaysia, Bayan Lepas, Penang, Malaysia; 2 Research Center for Applied Zoology, National Research and Innovation Agency (BRIN), Cibinong, Indonesia; 3 Department of Entomology, University of California, Riverside, CA, United States of America; University of South Dakota Sanford School of Medicine, UNITED STATES

## Abstract

The resurgence of bed bugs is a global phenomenon, but until now reports on bed bug infestations in Indonesia are rare. The success of bed bug control is affected by people’s knowledge and awareness. Stigma against bed bugs in Indonesia discourages the public from reporting infestations and therefore knowledge of their impact on public health is scarce. Herein, this study investigates the public’s perception, attitudes, and knowledge on bed bug-related issues in several major cities in Indonesia through an online survey. Despite low case reports, three in five respondents (n = 600) have encountered bed bugs at least once; mostly in their homes (74.1%). Approximately half of the respondents correctly identified bed bugs, whereas mites were often misidentified for bed bugs (26.3%). Bite marks were not a useful indicator for detecting bed bugs. We found age, gender, and level of education affects the public’s perception toward various bed bug-related issues. Regarding bed bug treatment, above 50% respondents are unaware of the availability of bed bug-specific insecticidal products and are unwilling to pay pest management professionals to control infestation. This study provides the first overview of the public’s awareness and perception of bed bug infestations in some major cities of Indonesia, which can be useful for designing public health policies for bed bug management. The reported data represents the perspectives of online users, most likely from metropolitan regions. A bigger monitoring program encompassing pest professionals and hospitality businesses would give a more thorough overview of the bed bug impact in Indonesia.

## Introduction

The association between bed bugs and humans can be traced back to ancient times. Although bed bugs arose approximately 115 mya, before their assumed hosts, bats, their association with humans comes much later [1]. Initially, the relationship between bed bugs and humans was believed to date back at least 3500 years after the discovery of bed bug fossils in Tell el-Amarna, Egypt [2]. However, a recent molecular study found that the association of bed bugs and human is estimated as early as human dispersal out of Africa during the last interglacial period [3,4]. Currently, two bed bug species are closely associated with humans, the tropical bed bug (*Cimex hemipterus*) and the common bed bug (*Cimex lectularius*). As modern bed bugs live closely with their human host, they spread readily as urbanization and trade expanded in the last few decades.

Bed bug infestations were a severe concern in the 17th and 18th centuries, particularly in England and the United States [5,6]. After World War II, bed bug infestations were well managed in most parts of the world partly due to the discovery and extensive use of synthetic insecticides such as DDT, pyrethroids, and malathion [7–9]. However, since the late 1990s, nearly every region of the world has experienced a global resurgence of bed bugs, including the United States [10], Europe [11], Asia [12], Australia [13], and Africa [14]. Two main aspects are assumed to be the major cause of this resurgence, an increase rate of global travel and insecticide resistance [15,16].

Bed bug cases are now rampant and can be found in most nations globally. The global resurgence of bed bugs causes financial losses to individuals and businesses in addition to being a significant nuisance to the public. Skin irritations are a common reaction from bed bug bites, ranging from a mild, itchy cutaneous sensation to more complex reactions, especially when secondary infection occurs [17]. In some cases, serious allergic reactions, including anaphylactic shock, can also occur in susceptible individuals [18,19]. Bed bug infestations can also cause psychological harm, such as anxiety disorders, insomnia, and post-traumatic stress disorder (PTSD) to the victim [20,21]. Apart from that, affected victims may suffer from expensive treatment costs and pest control services to eliminate bed bugs, in addition to the costs of replacing affected personal belongings [22]. Likewise, the resurgence of bed bug significantly impacted the hospitality and travel industries. The damages include loss of revenue, expensive eradication costs, legal costs for settlement over bed bug disputes, and brand damage [22,23].

To diminish the economic and public health burden imposed by bed bug infestations, various control and management strategies have been established. However, the success of any control strategy also relies on education and public awareness about the pest insect. For instance, the ability to recognize bed bug’s appearance and traces such as fecal spots and cast skin plays an important role in the detection of their infestation at an early stage, and thus enhances the efficacy of the control effort [24]. Therefore, public awareness campaigns concerning bed bugs are essential for preventing and controlling infestations. Thus far, studies on the public’s perception and awareness of bed bugs are limited, such as those reported in the United Kingdom [24], Germany [25], and Ethiopia [26].

Indonesia is the most populous and a highly urbanized Southeast Asian nation with more than 270 million residents. Indonesia, like the rest of the world, is vulnerable to a bed bug resurgence and the resulting economic losses. In neighboring countries within Southeast Asia, the resurgence and infestation of the tropical bed bug, *C*. *hemipterus* have been reported in Singapore, Malaysia, and Thailand [17,27,28]. Despite their importance, there have been no studies investigating public awareness and perception on bed bug infestations in Indonesia, and publications reporting the incidence of bed bug infestations are scarce [29]. The lack of reporting on bed bugs in Indonesia may give a false impression on the actual infestation levels of this pest in the country. Reporting the presence of bed bugs can be hampered by social stigma, whereby an afflicted person may be associated with being poor and of low social status or living in unsanitary conditions, which is often not the case [30]. Thus, this causes the public to shy away from reporting infestations and the impact on their health and refuse to seek assistance from professionals [30]. This study’s objective is to evaluate public knowledge, attitudes, and perceptions of bed bug infestation and management in a sample of Indonesian urban regions using online surveys. The online method may be advantageous in overcoming the stigma associated with reporting a bed bug encounter because it was fully anonymous.

## Materials and methods

### Study limitations

This study was conducted online through social media platforms due to travel and movement restrictions during the Community Activities Restrictions Enforcement (CARE) in Indonesia because of the worsening COVID-19 pandemic at the time of the study. The results of this study therefore reflect the views and knowledge of Indonesian online users, most likely those from urban areas, and may not necessarily reflect the country’s entire population. Even though only a portion of the population was considered, the study’s findings, which were previously unavailable, provide a critical overview of Indonesian opinions on bed bug infestations and control options from several major cities.

### Data collection

The survey was conducted through an online questionnaire, which was developed using an online platform, Google Form (https://bit.ly/3aLldce) (S1 dataset). The study took place from May to October 2021. The questionnaire was presented in both Bahasa Indonesia (primary) and English (translated). Wherever there is a discrepancy, the original meaning in Bahasa Indonesia is used as the correct version. The purpose of the survey and guidelines for answering the questionnaire were stated at the beginning of the questionnaire. The link for the online survey was shared with the public through various social networking platforms with public access, such as university and research-related Facebook groups, Instagram, and Twitter accounts. This study was conducted using an epidemiological approach with undisclosed personal data and the identity of the respondents. As the online survey was conducted completely anonymous through public platforms and bed bug is a global pest (i.e., not endemic to any region), ethical approval is not required under the Indonesia’s regulation.

The questionnaire was divided into five main sections. The first section contains information about the respondent’s demographic characteristics such as gender, age, residency, and level of education. The second section inquires respondents’ past experience with bed bugs and their ability to recognize the bed bugs. This includes multiple-choice questions containing five different images of insects (including bed bugs) to test their ability to identify the bed bugs correctly. Respondents who answered "Yes" to the statement "I have seen a bed bug" but selected the incorrect image of the insect were considered to have misidentified the bed bug. The third section is concerned with responses to the bed bug’s bite, such as whether respondents have been bitten by the bed bug and whether they can distinguish the bed bug’s bite from other insect’s bite. The fourth section was related to the respondents’ knowledge about places that can be infested by bed bugs other than their own home. The last section questions the respondents’ knowledge and experience in getting rid of the bed bugs. In this section, respondents’ preferences, such as a do-it-yourself (DIY) approach or requesting pest control operator (PCO) intervention, were solicited. In sections three to five, we also incorporated the Likert scale from 1 (strongly disagree) to 5 (strongly agree) to assess the respondents’ perceptions on each of the topics presented therein.

### Statistical analysis

Respondents’ demographic characteristics and questions with yes or no answers were analyzed as percentages. Meanwhile, Likert scale responses were presented as frequencies. The Kruskal-Wallis H test was used to analyze the influence of different age groups on their experience in encountering bed bug infestations. For binary questions (with yes-no answers), Fisher’s exact test was used to determine statistical significance between the two groups. For Likert scale responses, we divided the respondents into two groups based on three categories: i) education background (those with less than a bachelor’s degree and the remaining respondents), ii) gender (male and female respondents), and iii) age (respondents below the age of 30 and 30 years old and above). The age groups were divided based on the middle point of six age groups intervals in the survey form (Table 1). Descriptive statistics and Mann-Whitney U test were used to test for any significant differences between these respective groups in Likert scale responses. All statistical tests were conducted in SPSS version 26 for Windows (IBM Corp., Armonk, NY, USA) at the alpha significance level of 0.05.

**Table 1:** Online survey responses.

No	Variable (n)	Weighted Freq.	%
**Section 1: Demographics of the respondents**			
**1**	**Gender (600)**		
	Female	339	56.5%
	Male	261	43.5%
**2**	**Age (600)**		
	15–20	47	7.8%
	21–25	49	8.2%
	26–30	85	14.2%
	31–40	215	35.8%
	41–50	116	19.3%
	> 50	88	14.7%
**3**	**Highest level of education (600)**		
	SD / Primary School	0	0.0%
	SMP / Junior High School	1	0.2%
	SMA / High School	38	6.3%
	D3 / Diploma	36	6.0%
	S1 / Bachelor Degree	238	39.7%
	S2 / Master Degree	178	29.7%
	S3 / Doctoral Degree	109	18.2%
**Section 2: Encountering and recognizing bed bug**			
**4**	**I have encountered bed bugs (600)**		
	Yes	362	60.3%
	No	238	39.7%
**5**	**When did you encounter the bed bugs (361)**		
	2021—present	30	8.3%
	2011–2020	121	33.5%
	2001–2010	72	19.9%
	1991–2000	60	16.6%
	< 1990	78	21.6%
**6**	**Where did you encounter the bed bugs (355)**		
	House	263	74.1%
	Hotel	59	16.6%
	Cinema	21	5.9%
	Public transport	30	8.5%
	Hostel	24	6.8%
	Hospital	1	0.3%
	School	1	0.3%
	Public area	1	0.3%
**7**	**How many times did you encounter the bed bugs (359)**		
	1–2	157	43.7%
	3–5	80	22.3%
	> 5	122	34.0%
**8**	**I know the shape or form of bed bugs (600)**		
	Yes	407	67.8%
	No	193	32.2%
**9**	**Which one these is the bed bug from the 5 pictures below?**		
	Tick	68	11.3%
	Mite	158	26.3%
	Mosquito	3	0.5%
	Ant	2	0.3%
	Bed bug	369	61.5%
**10**	**Number of respondents correctly identified the bed bug (from the respondents that claimed they know bed bug appearance) (407)**		
	Correct	297	73.0%
	Wrong	110	27.0%
**Section 3: Responses to bed bug’s bites**			
**11**	**I can differentiate bed bug’s bite apart from those of other insects (600)**		
	Strongly Disagree	128	21.3%
	Disagree	165	27.5%
	Neutral	145	24.2%
	Agree	103	17.2%
	Strongly Agree	59	9.8%
**12**	**I know that bed bug’s bite can cause itchiness and rash (600)**		
	Strongly Disagree	22	3.7%
	Disagree	32	5.3%
	Neutral	92	15.3%
	Agree	205	34.2%
	Strongly Agree	249	41.5%
**13**	**I have been or knew someone who has been bitten by bed bugs and had an allergic reaction (600)**		
	Yes	408	68.0%
	No	192	32.0%
	Strongly Agree	29	4.8%
**14**	**I have been or knew someone who has been bitten by bed bugs in public places (600)**		
	Yes	120	20.0%
	No	480	80.0%
**Section 4: Bed bug infestation in public places**			
**15**	**Apart from home, bed bugs can also infest public places such as hotel, cinemas, and public transportation (600)**		
	Strongly Disagree	7	1.2%
	Disagree	14	2.3%
	Neutral	84	14.0%
	Agree	201	33.5%
	Strongly Agree	294	49.0%
**16**	**Every time I go to a public place (e.g., hotel, office, public transportation) I will check for bed bugs (600)**		
	Strongly Disagree	61	10.2%
	Disagree	144	24.0%
	Neutral	204	34.0%
	Agree	107	17.8%
	Strongly Agree	84	14.0%
**17**	**I find bed bug infestation at home and in public places as annoying (600)**		
	Strongly Disagree	3	0.5%
	Disagree	6	1.0%
	Neutral	45	7.5%
	Agree	131	21.8%
	Strongly Agree	415	69.2%
**Section 5: Responses towards management of bed bugs**			
**18**	**I have looked for information on ways to control bed bug infestation (600)**		
	Yes	245	40.8%
	No	355	59.2%
**19**	**I know that there are insecticides specific for bed bugs available (600)**		
	Yes	242	40.3%
	No	358	59.7%
**20**	**I know there are local companies that offer bed bug control services (600)**		
	Yes	278	46.3%
	No	322	53.7%
**21**	**I have asked the services from pest control operators for inspection and controlling bed bugs at my house (for respondents that chose yes for question4) (362)**		
	Yes	23	6.4%
	No	339	93.6%
**22**	**When there is a bed bug infestation in my house, I will ask pest control operator for help (600)**		
	Strongly Disagree	73	12.2%
	Disagree	147	24.5%
	Neutral	208	34.7%
	Agree	91	15.2%
	Strongly Agree	81	13.5%
**23**	**I would rather do it by myself to control bed bug infestation at my house instead of calling for pest control operators because it is more economical (600)**		
	Strongly Disagree	32	5.3%
	Disagree	69	11.5%
	Neutral	109	18.2%
	Agree	229	38.2%
	Strongly Agree	161	26.8%
**24**	**I’m going to find out more about companies that offer bed bug control services (600)**		
	Strongly Disagree	39	6.5%
	Disagree	96	16.0%
	Neutral	201	33.5%
	Agree	151	25.2%
	Strongly Agree	113	18.8%

## Results

### Demographics of the respondents

**Fig 1:**
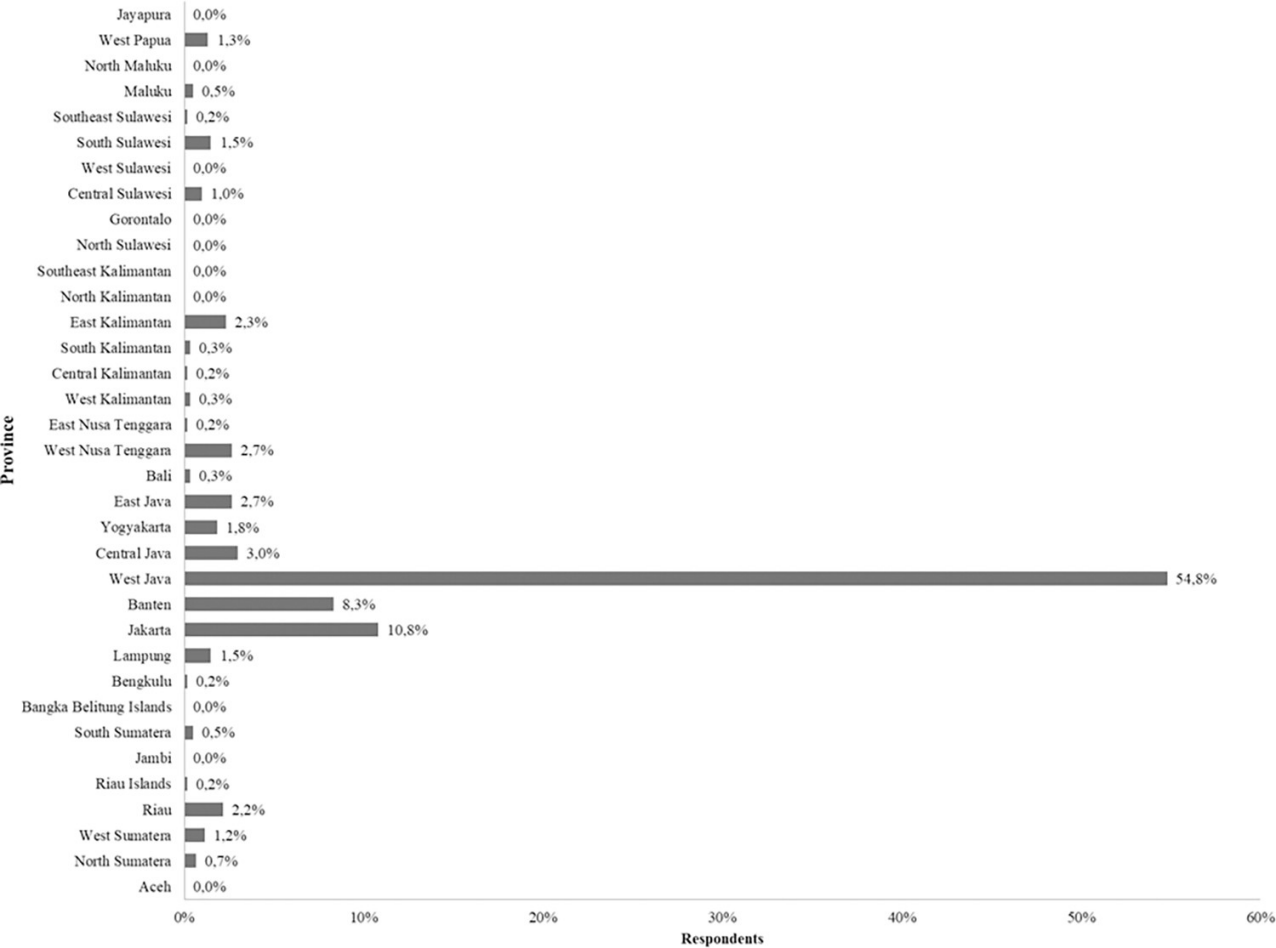
Percentage of respondents based on their residency.

The online survey received 600 responses, with nearly similar numbers of female (56.5%) and male (43.5%) respondents (Table 1, question 1). The largest age group (35.8%) was between the ages of 31 and 40, followed by those between the ages of 41 and 50 (19.3%) (Table 1, question 2). The survey received responses from 25 provinces out of 35 provinces in Indonesia. Residency of the respondents were mostly from Java Island, dominated by West Java (54.8%), followed by Jakarta (10.8%), and Banten (8.5%) (Fig 1). Most of the respondents were reported to have a bachelor’s degree (39.7%), followed by those with a master’s degree (29.7%) (Table 1, question 4).

### Encountering and recognising bed bugs

**Fig 2:**
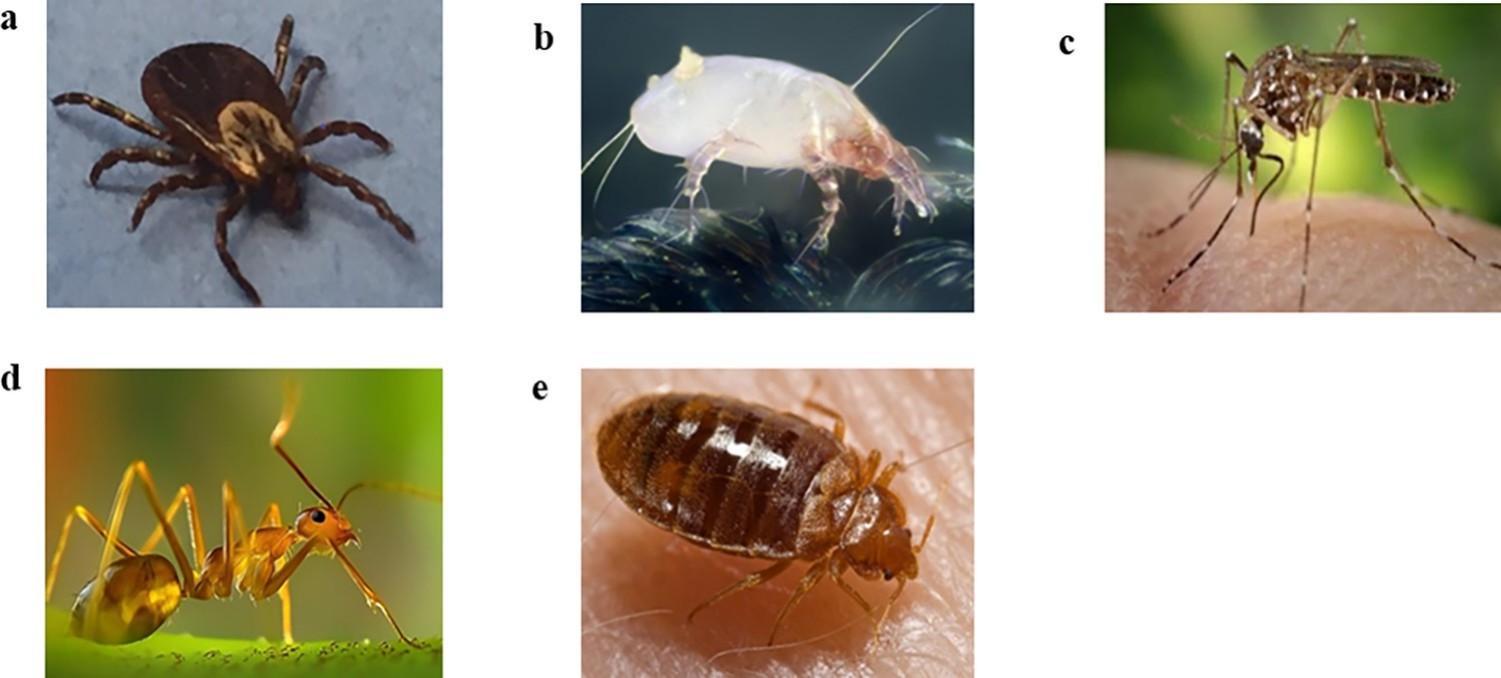
Pictures of arthropods used for the online survey to evaluate respondent’s ability to identify bed bug. (a) Tick (National Institute of Allergy and Infectious Disease 2017, https://www.nih.gov/news-events/news-releases/tickborne-diseases-are-likely-increase-say-nih-officials, CC-BY-NC). (b) Mite (Gilles S Martin 2010, https://commons.wikimedia.org/wiki/File:House_dust_mite_%28Dermatophagoides_pteronyssinus%29.jpg, CC-BY-SA). (c) Mosquito (Frank H Collins 2006, https://theconversation.com/global-warming-to-expose-more-people-to-zika-spreading-mosquito-aedes-aegypti-58908, CC-BY-NC). (d) Ant (Abdulmominbd 2015, https://commons.wikimedia.org/wiki/File:The-Ant.jpg, CC-BY-SA). (e) Bed bug (CDC 2006, https://commons.wikimedia.org/wiki/File:Bed_bug,_Cimex_lectularius.jpg, CC-BY).

**Fig 3:**
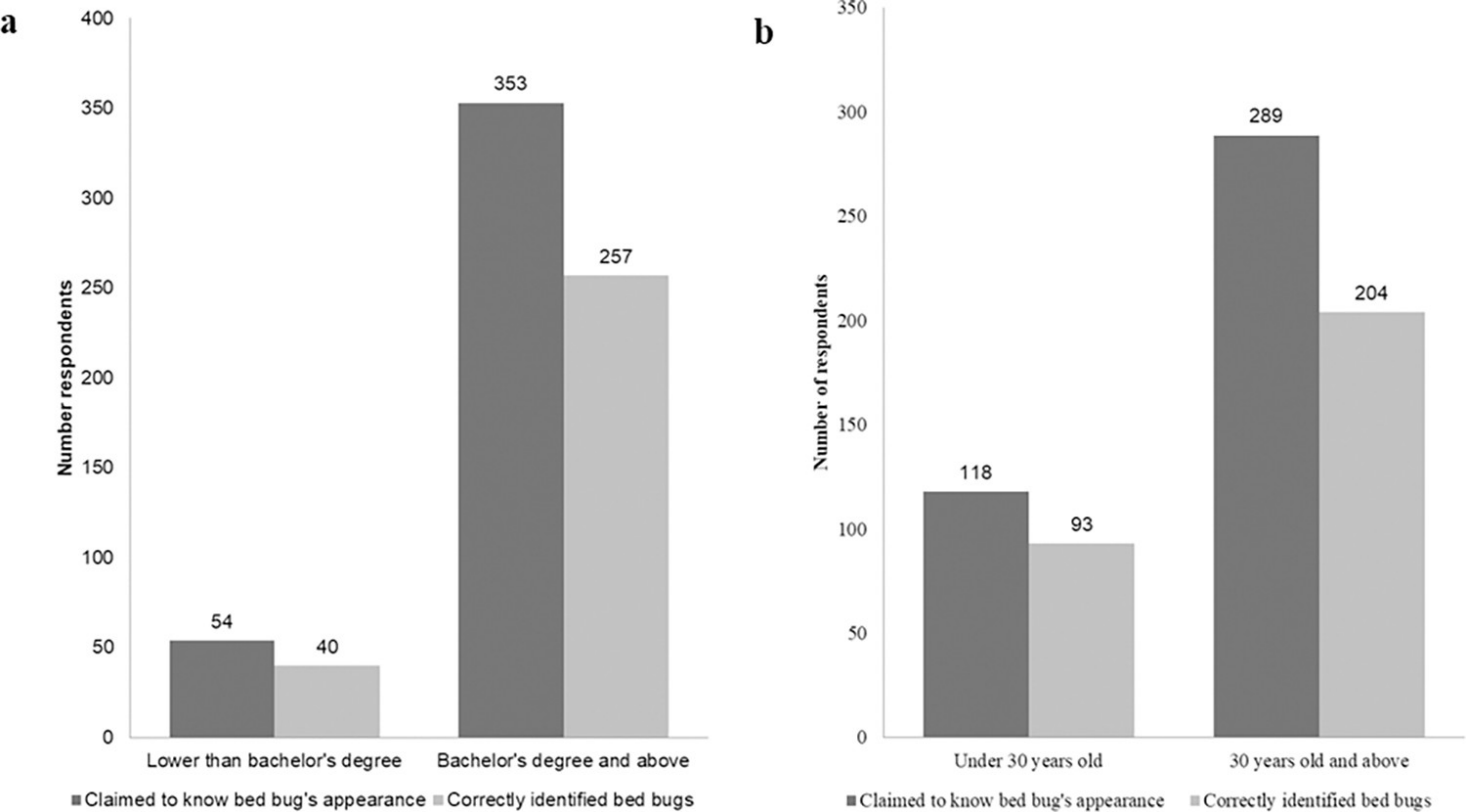
Correlation between respondents’ (a) education background (Fisher’s exact test, p = 1.000) and (b) age (Fisher’s exact test, p = 0.110) in their ability to identify bed bugs.

Of the 600 respondents, 362 said that they have encountered bed bugs (Table 1, question 4). The participants were then asked about their encounters with bed bugs, including where, when, and how often they had seen them (Table 1, questions 5–7). Around 33.5% of respondents reported encountering bed bugs between 2011 and 2020, followed by 21.6% who claimed to have seen them before 1990 (this data only applies to those born before 1990) and 19.0% who reported encounters between 2001 and 2010 (Table 1, question 5). More specifically, respondents born after 1990 (n = 89) reported seeing bed bugs most frequently between 2011 and 2020 (43.33%), followed by 2001 to 2010 (18.89%). Those born prior to 1990 (n = 273), on the other hand, reported seeing bed bugs both between 2011 and 2020 (25.27%) and prior to 1990 (23.08%). Most of them encountered bed bugs in their home (74.1%), followed by hotels (16.6%), and public transport (8.5%) (Table 1, question 6). When it comes to the frequency of encountering bed bugs, 43.7% of respondents said they had seen them at least once or twice (Table 1, question 7). Interestingly, 34.0% (Table 1, question 7) of respondents had encountered bed bugs more than five times, showing that bed bug infestations were frequent but likely underreported in Indonesia due to a lack of reporting platforms, and the value of reporting them appeared secondary to other public health surveillance such as vector-borne diseases [31]. In addition, those born before 1990 (35.53%) are more likely to have experienced bed bugs more than five times than those born after 1990 (28.09%). Among the 88 individuals older than 50, 81.0% reported having experienced bed bug infestations. This represents the greatest proportion of all age groups (Kruskal-Wallis H(5) = 27.74, p < 0.001). From the 600 people who answered the survey, 67.8% (Table 1, question 8) said they could recognize a bed bug by its appearance. However, only 73.0% of those 67.8% (Table 1, question 10) were able to correctly identify the image of a bed bug when it was shown alongside images of ticks, mites, mosquitoes, and ants (Fig 2). Our findings also revealed that approximately a quarter of respondents (26.3%) misidentified bed bugs for mites (Table 1, question 9). The lack of a scale in the images provided may have also contributed to the misidentification. The ability to distinguish bed bugs does not differ significantly between respondents aged 30 and older (n = 273) and those aged under 30 (n = 89) (Fisher’s exact test, p = 0.110) (Fig 3). We also found that there is no difference in the ability to distinguish bed bug from other arthropod species between respondents with a higher education level (i.e., with at least a bachelor’s degree) (n = 257) and respondents with a lower education background (n = 40) (Fisher’s exact test, p = 1.000) (Fig 3).

### Responses to bed bug bites

The majority of respondents (75.7%) concurred that bed bug bites can result in redness and itchiness (Table 1, question 12) and 68.0% of the respondents claimed that they have been bitten or knew someone who has been bitten by bed bugs and had an allergic reaction (Table 1, question 13). However, 73.0% of participants were either unsure (neutral response) or unable to distinguish a bed bug bite from other insect bites (Table 1, question 11). This shows that bite marks are not a reliable indicator of the presence of bed bugs, and that it may be necessary to view the insects in person to be certain. Twenty percent of the respondents stated that they have been bitten or knew someone who has been bitten by bed bugs in public places (Table 1, question 14).

Although overall respondents are generally poor at recognizing bed bug bite marks from other arthropods, male respondents are relatively more likely than female respondents to distinguish bed bug bites from other arthropods (Mann-Whitney U = 38593.50, p = 0.006) (Table 2). Similarly, respondents without a bachelor’s degree showed an overall higher positive attitude to distinguish bed bug bites from those of other arthropods than those with higher education background (Mann-Whitney U = 18747.50, p = 0.049) (Table 2). However, there was no significant differences between the age groups in their response to identifying the bed bug’s bite (Mann-Whitney U = 36104.00, p = 0.339) (Table 2). Respondents across all demographics (gender, education level, and age) are equally aware that bed bug bites can cause itchiness and rashes (Table 2).

**Table 2:** Perception, attitude, and knowledge on bed bug’s bite, infestation, and management among the respondents based on gender, education background, and age.

No	Question/Category		N	Likert Scale										Total Score	Mean Likert Score ± SD	Mann-Whitney U	Z-score	*p*-value
				Strongly disagree (1)		Disagree (2)		Neutral (3)		Agree (4)		Strongly agree (5)						
				F	%	F	%	F	%	F	%	F	%					
1	**I can differentiate bed bug’s bite apart from those of other insects**																	
	**Gender**	Female	339	81	23.89%	97	28.61%	87	25.66%	44	12.98%	30	8.85%	862	2.543 ± 1.233	38593.50	-2.753	0.006
		Male	261	47	18.01%	68	26.05%	58	22.22%	59	22.61%	29	11.11%	738	2.828 ± 1.276			
	**Education Background**	Without a bachelor’s degree	75	13	17.33%	20	26.67%	22	29.33%	13	17.33%	7	9.33%	206	2.747 ± 1.267	18747.50	-0.687	0.0492
		Bachelor’s degree and above	525	115	21.90%	145	27.62%	123	23.43%	90	17.14%	52	9.90%	1394	2.655± 1.210			
	**Age**	Under 30 years old	181	38	20.99%	56	30.94%	44	24.31%	29	16.02%	14	7.73%	468	2.586 ± 1.206	36104.00	-0.956	0.339
		30 years old and above	419	90	21.48%	109	26.01%	101	24.11%	74	17.66%	45	10.74%	1132	2.702 ± 1.281			
2	**I know that bed bug’s bite can cause itchy and rash**															** **	** **	
	**Gender**	Female	339	14	4.13%	20	5.90%	51	15.04%	108	31.86%	146	43.07%	1369	4.038 ± 1.091	43664.50	-0.29	0.772
		Male	261	8	3.07%	12	4.60%	41	15.71%	97	37.16%	103	39.46%	1058	4.054 ± 1.006			
	**Education Background**	Without a bachelor’s degree	75	2	2.67%	4	5.33%	9	12.00%	26	34.67%	34	45.33%	311	4.147± 1.009	18485.50	-0.91	0.363
		Bachelor’s degree and above	525	20	3.81%	28	5.33%	83	15.81%	179	34.10%	215	40.95%	2116	4.030± 1.061			
	**Age**	Under 30 years old	181	10	5.52%	9	4.97%	26	14.36%	61	33.70%	75	41.44%	725	4.006 ± 1.123	37492.50	-0.233	0.816
		30 years old and above	419	12	2.86%	23	5.49%	66	15.75%	144	34.37%	174	41.53%	1702	4.062 ± 1.024			
3	**Apart from home. bed bugs can also infest public places such as hotel. cinemas. and public transportation**															** **	** **	
	**Gender**	Female	339	4	1.18%	10	2.95%	46	13.57%	101	29.79%	178	52.51%	1456	4.295 ± 0.895	41498.00	-1.419	0.156
		Male	261	3	1.15%	4	1.53%	38	14.56%	100	38.31%	116	44.44%	1105	4.234 ± 0.838			
	**Education Background**	Without a bachelor’s degree	75	1	1.33%	2	2.67%	9	12.00%	22	29.33%	41	54.67%	325	4.333 ± 0.890	18495.00	-0.925	0.355
		Bachelor’s degree and above	525	6	1.14%	12	2.29%	75	14.29%	179	34.10%	253	48.19%	2236	4.259 ± 0.868			
	**Age**	Under 30 years old	181	2	1.10%	7	3.87%	29	16.02%	68	37.57%	75	41.44%	750	4.144± 0.901	33500.50	-2.471	0.013
		30 years old and above	419	5	1.19%	7	1.67%	55	13.13%	133	31.74%	219	52.27%	1811	4.322 ± 0.852			
4	**Every time I go to a public place (e.g. hotel. office. public transportation) I will check for bed bugs**															** **	** **	
	**Gender**	Female	339	33	9.73%	72	21.24%	127	37.46%	65	19.17%	42	12.39%	1028	3.032 ± 1.137	42869.50	-0.672	0.501
		Male	261	28	10.73%	72	27.59%	77	29.50%	42	16.09%	42	16.09%	781	2.992 ± 1.231			
	**Education Background**	Without a bachelor’s degree	75	7	9.33%	17	22.67%	20	26.67%	14	18.67%	17	22.67%	242	3.227± 1.290	17612.00	-1.527	0.127
		Bachelor’s degree and above	525	54	10.29%	127	24.19%	184	35.05%	93	17.71%	67	12.76%	1567	2.985 ± 1.159			
	**Age**	Under 30 years old	181	23	12.71%	49	27.07%	57	31.49%	34	18.78%	18	9.94%	518	2.862 ± 1.163	34106.00	-2.021	0.043
		30 years old and above	419	38	9.07%	95	22.67%	147	35.08%	73	17.42%	66	15.75%	1291	3.081 ± 1.179			
5	**I find bed bug infestation at home and in public places as annoying**															** **	** **	
	**Gender**	Female	339	1	0.29%	2	0.59%	23	6.78%	64	18.88%	249	73.45%	1575	4.646 ± 0.661	39821.00	-2.587	0.010
		Male	261	2	0.77%	4	1.53%	22	8.43%	67	25.67%	166	63.60%	1174	4.498 ± 0.778			
	**Education Background**	Without a bachelor’s degree	75	2	2.67%	0	0.00%	3	4.00%	10	13.33%	60	80.00%	351	4.680 ± 0.791	17345.00	-2.056	0.040
		Bachelor’s degree and above	525	1	0.19%	6	1.14%	42	8.00%	121	23.05%	355	67.62%	2398	4.568 ± 0.706			
	**Age**	Under 30 years old	181	3	1.66%	2	1.10%	11	6.08%	33	18.23%	132	72.93%	832	4.597 ± 0.794	36103.00	-1.149	0.251
		30 years old and above	419	0	0.00%	4	0.95%	34	8.11%	98	23.39%	283	67.54%	1917	4.575± 0.682			
6	**When there is a bed bug infestation in my house. I will ask pest control operator for help**															** **	** **	
	**Gender**	Female	339	31	9.14%	80	23.60%	117	34.51%	58	17.11%	53	15.63%	1039	3.065 ± 1.182	38063.50	-3.033	0.002
		Male	261	42	16.09%	67	25.67%	91	34.87%	33	12.64%	28	10.73%	721	2.762 ± 1.185			
	**Education Background**	Without a bachelor’s degree	75	18	24.00%	13	17.33%	28	37.33%	9	12.00%	7	9.33%	199	2.653 ± 1.236	16999.00	-1.979	0.048
		Bachelor’s degree and above	525	55	10.48%	134	25.52%	180	34.29%	82	15.62%	74	14.10%	1561	2.973 ± 1.182			
	**Age**	Under 30 years old	181	19	10.50%	43	23.76%	51	28.18%	36	19.89%	32	17.68%	562	3.105 ± 1.249	33752.50	-2.21	0.027
		30 years old and above	419	54	12.89%	104	24.82%	157	37.47%	55	13.13%	49	11.69%	1198	2.859 ± 1.160			
7	**I would rather do it by myself to control bed bug infestation at my house instead of calling for pest control operators because it is more economical**															** **	** **	
	**Gender**	Female	339	23	6.78%	49	14.45%	75	22.12%	103	30.38%	89	26.25%	1203	3.549 ± 1.214	37487.50	-3.349	0.001
		Male	261	9	3.45%	20	7.66%	34	13.03%	126	48.28%	72	27.59%	1015	3.889 ± 1.007			
	**Education Background**	Without a bachelor’s degree	75	1	1.33%	9	12.00%	8	10.67%	23	30.67%	34	45.33%	305	4.067 ± 1.082	15202.50	-3.334	0.001
		Bachelor’s degree and above	525	31	5.90%	60	11.43%	101	19.24%	206	39.24%	127	24.19%	1913	3.644 ± 1.139			
	**Age**	Under 30 years old	181	12	6.63%	19	10.50%	31	17.13%	70	38.67%	49	27.07%	668	3.691 ± 1.171	37773.00	-0.078	0.937
		30 years old and above	419	20	4.77%	50	11.93%	78	18.62%	159	37.95%	112	26.73%	1550	3.699 ± 1.128			
8	**I’m going to find out more about companies that offer bed bug control services**															** **	** **	
	**Gender**	Female	339	15	4.42%	45	13.27%	108	31.86%	97	28.61%	74	21.83%	1187	3.501 ± 1.105	36163.50	-3.967	< 0.001
		Male	261	24	9.20%	51	19.54%	93	35.63%	54	20.69%	39	14.94%	816	3.126 ± 1.165			
	**Education Background**	Without a bachelor’s degree	75	10	13.33%	12	16.00%	27	36.00%	14	18.67%	12	16.00%	231	3.080 ± 1.239	17068.00	-1.929	0.054
		Bachelor’s degree and above	525	29	5.52%	84	16.00%	174	33.14%	137	26.10%	101	19.24%	1772	3.375 ± 1.128			
	**Age**	Under 30 years old	181	6	3.31%	31	17.13%	56	30.94%	46	25.41%	42	23.20%	630	3.481 ± 1.123	34461.50	-1.835	0.067
		30 years old and above	419	33	7.88%	65	15.51%	145	34.61%	105	25.06%	71	16.95%	1373	3.277 ± 1.151			

### Bed bug infestations in public places

In this section, respondents were asked for their opinion regarding bed bug infestations in public spaces. Most of the respondents (82.5%) agree that infestation can occur in public places, such as hotels, cinemas, and public transportation (Table 1, question 15). They also agree (91.0%) that bed bug outbreaks in public spaces are vexing (Table 1, question 17). Nonetheless, when asked if they would check for bed bugs when they are in public places, we received mixed responses, with most being indifferent to check for bed bugs every time they go to public places (68.2%) (Table 1, question 16).

Both male and female participants were equally aware that bed bug infestations can occur both within the home and in public settings (Mann-Whitney U = 41498.00, p = 0.156), and their opinions were not influenced by their educational levels (Mann-Whitney U = 18495.00, p = 0.355) (Table 2). Nonetheless, respondents aged 30 and older are more aware than those aged under 30 that public venues can be infested with bed bugs (Mann-Whitney U = 33500.50, p = 0.013) (Table 2). Similarly, adults aged 30 and above have a higher positive response in terms of willingness to check for bed bugs in public places than the younger respondents (Mann-Whitney U = 34106.00, p = 0.043), although all age groups feel that bed bug infestations are annoying both at home and in public places (Mann-Whitney U = 36103.00, p = 0.251) (Table 2).

Gender (Mann-Whitney U = 42869.50, p = 0.501) and level of education (Mann-Whitney U = 17612.00, p = 0.127) had no effect on respondents’ willingness to check for bed bugs every time they are in public places (Table 2). On average, female respondents were less tolerant of bed bug infestations at home and in public places when compared with male respondents (Mann-Whitney U = 39821.00, p = 0.010) (Table 2). Additionally, respondents with at least a bachelor’s degree felt that bed bug infestations at home and in public areas are less annoying than respondents with a lower educational level (Mann-Whitney U = 17345.00, p = 0.040) (Table 2), probably because the social stigma associated with bed bugs as a sign of poor hygiene is less common among the groups with a higher academic background.

### Responses towards management of bed bugs

Respondents’ knowledge and awareness toward the management of bed bugs were assessed through various types of questions. When asked if they had looked up for information on how to control bed bug infestations, 59.2% of the respondents answered no (Table 1, question 18). More than half of the respondents (59.7%) were unaware that insecticides for controlling bed bugs were available on the market (Table 1, question 19). Additionally, over half of the respondents (53.7%) were uninformed about the existence of local companies that provide bed bug control services (Table 1, question 20). Only 6.4% of 362 people claimed to have contacted pest control professionals (PCOs) after discovering bed bugs in their residences (Table 1, question 21). Because it is more affordable to treat bed bug infestations on their own, the majority of respondents (65.0%) opted not to engage pest control services if their homes were infested with bed bugs (Table 1, questions 22–23). Furthermore, more than half of the respondents (56%) are still hesitant to find out more about pest control solutions for dealing with bed bug infestations (Table 1, question 24).

We found that female respondents are more likely than male respondents to call pest control operators (PCOs) when bed bugs infest their home (Mann-Whitney U = 38063.50, p = 0.002) (Table 2). Respondents with at least a bachelor’s degree were more likely than others to request pest control operator assistance (Mann-Whitney U = 16999.00, p = 0.048) (Table 2). This is because respondents with an education lower than a bachelor’s degree also found that it is more economical to control bed bugs on their own than calling the PCOs when compared with those with a bachelor’s degree or higher (Mann-Whitney U = 15202.50, p = 0.001) (Table 2). In addition, respondents aged under 30 years old are more willing to call for PCOs for managing bed bugs than older respondents (Mann-Whitney U = 33752.00, p = 0.027) (Table 2).

Male respondents, on the other hand, prefer to do their own bed bug control rather than calling PCOs because they found it as a less expensive option when compared with females. (Mann-Whitney U = 37487.50, p = 0.001) (Table 2). On the other hand, age had no effect in respondents’ preference whether to control bed bugs by themselves or calling PCOs (Mann-Whitney U = 36245.00, p = 0.370) (Table 2). Aside from that, female respondents are more likely than males to be willing to seek more information about pest control services (Mann-Whitney U = 36163.50, p < 0.001) (Table 2). Age (Mann-Whitney U = 34461.50, p = 0.067) and education background (Mann-Whitney U = 17068.00, p = 0.054) have no significant influence on the willingness of the respondents to seek additional information on pest control services (Table 2).

## Discussion

Bed bug infestations have become prominent worldwide, generating increased public concern. Although there are insufficient evidence to connect bed bugs and disease transmission, the clinical consequences of their bite cannot be ignored [7,27]. In addition, bed bug infestations are often costly to manage [32]. Studies on the public’s view and understanding of bed bug problems are scarce, despite the availability of surveys regarding bed bugs based on clinical [33,34] and income perspectives [35,36]. This survey was conducted online due to travel and movement limitations imposed by the Community Activities Restrictions Enforcement (CARE) in Indonesia at the time of the study due to the escalating COVID-19 outbreak. We recognize that our results could be impacted by the biases and constraints of the online platform. Notably, the respondents in this study were primarily college educated, which is likely owing to the online survey, as college educated people are more inclined to participate in internet surveys. Despite the differences in numbers, we find significant differences in the views of respondents with at least a bachelor’s degree versus other respondents for some of the survey responses (Table 2), indicating that the survey adequately represents the different education demographic. Aside from that, this study, however, provides the first evaluation of the bed bug infestation issue in several major cities, particularly Java Island in Indonesia, and the public’s perception of it. Bed bug cases in Asia (Japan, China, and Southeast Asia) were common from the 1940s to the 1960s, before the number of cases declined in the 1970s and 1980s [17,37]. Subsequently, the resurgence of bed bugs in Asia was documented from the late 1990s to the early 2000s after a three-decade hiatus [17,37]. According to our study, bed bug cases in some of Indonesia’s major cities, particularly West Java, Jakarta, and Banten, followed a similar pattern, with a significant proportion of cases occurring before the 1990s and the resurgence steadily increasing from 2001 to 2010 and becoming more noticeable from 2011 to 2020. The likelihood of encountering bed bugs is generally higher in the older age group (> 50 years old). Similarly, a study in Germany also indicated that people who are older than 60 years have better knowledge about bed bugs than other age groups [25]. This age group likely has a high chance of encountering bed bugs because they have experienced both the pre- and post-resurgence stages. The ability to recognize bed bugs is vital for early detection of their infestation [38], as misidentification frequently leads to ineffective pest management and unnecessary costs [39]. However, public awareness of bed bugs varies by region of the world. In this study, the public’s awareness of bed bugs in Indonesia (73.0%, n = 600) was found higher than in the United Kingdom (10.0%, n = 358) [24] and Germany (13.0%, n = 391) [25], but it was lower when compared with Ethiopia (91.6%, n = 260) [26]. A review by Schoelitsz et al. [39] described that the ability to accurately identify household insect pests depends on a prior encounter or negative experience with the pests. Thus, the low level of bed bug awareness in Europe (the United Kingdom and Germany) when compared with Indonesia and Ethiopia could be ascribed to a lower level of bed bug infestation rate in that region. Furthermore, bed bug infestation could have been kept under control in Europe due to the availability and application of the code of practice and guidelines for bed bug management. Developed countries generally have their own standards for bed bug management, for example, Europe has the *European Code of Practice for Bed Bug Management* [40,41], Australia has *A Code Practice for the Control of Bed Bug Infestation in Australia* [42], and the United States has the *NPMA Bed Bugs Best Management Practices* [43]. To our knowledge, a similar standard is not available in Asia and Africa. The fact that most of the respondents in this study are from Java Island may also contribute to the high knowledge of bed bugs among the respondents. Java is Indonesia’s urbanization epicenter, with a high mobility of people coming in and out [44], raising the risk of bed bug exposure and infestation. Aside from that, differences in financial resources and people’s mindsets among nations may also contribute to the observed discrepancies. Poverty and poor pest management practices, for instance, may be to blame for Ethiopia’s high prevalence of bed bug exposure and infestation [19,26,45].

The detection of bed bug bite marks can be an early indicator of bed bug infestation in a unit, however, it is very difficult, if not impossible, to distinguish bed bug bites from other insects [46]. In this study, more than half of the participants claimed to have experienced an allergic reaction after being bitten by bed bugs or knowing someone who did. This also implies that a considerable proportion of people do not notice bites or have bites but do not experience symptoms. Nonetheless, this data should be interpreted with caution as the respondents could easily mistake other insect bites for bed bug bite marks or vice versa. Moreover, reactions to a bed bug’s bite vary among people. The most commonly reported skin reaction is a faint bite mark with little redness that often goes unnoticed [47]. Correspondingly, the majority of participants in this survey believe they are unable to tell a bed bug bite from a bite from another insect, while being aware that a bed bug bite might result in a red rash and itchiness. Although educating the public on bite symptoms of bed bug may be difficult, conducting training for healthcare personnel is nonetheless essential [46]. Delayed diagnosis or misdiagnosis will result in incorrect therapy and repeated bites due to control failure [46,48]. As a result, the effect of the bites becomes more severe and difficult to be treated [8,46]. Instead, accurate diagnosis of the bite marks showed positive effects on the management and control of bed bugs [46].

Although bed bug infestations are most common in homes, they can also be found in public places such as hotels, hospitals, schools, public transport, nursing homes, office buildings, dormitories, and movie theaters [17,20,29,34,49]. In line with this, we found that most respondents were aware that bed bug infestations can occur in public places and are disturbed by their presence. Nonetheless, as indicated by the survey, people are less likely to check for bed bugs in public places since chances of encountering bed bugs in public places can be rare and happens by chance. Nonetheless, public places do pose a substantial risk as a source for picking up bed bugs and transferring them to someone’s home or another location [20]. This is especially true because bed bugs can readily infest new places by hitchhiking into people’s belongings such as luggage or clothes [50]. Therefore, being vigilant and prepared to check for bed bugs in public spaces that have a higher likelihood for encountering bed bugs such as hotel rooms may help to stop the pest from spreading and infesting other locations.

There are many approaches to eliminate bed bug infestations, including chemical and non-chemical applications [51,52]. However, it requires adequate knowledge to choose an appropriate method to eliminate bed bug infestations. We discovered that most of the respondents had little knowledge on how to eliminate the bed bugs and unaware of control options to manage the pest. The respondents prefer to eliminate bed bug infestations on their own because they believe it will be cost-effective. This is due to the perception that PCO service is often costly. Similarly, a surveillance study done by the PCOs in 2010 in the United States, revealed that the respondents prefer to choose a “Do-It-Yourself (DIY)” control option before calling the pest control operator for assistance [49]. Several factors were suggested might be affecting their preference for a DIY approach to other methods when handling bed bugs. These include misidentification of bed bugs, embarrassment, and costly treatment [49,53]. Interestingly, besides bed bugs, Indonesians also have poor awareness of mosquito control. For example, a study in Central Java involving 273 respondents showed a lack of awareness and poor understanding on space spraying to eliminate mosquitoes in an effort to fight dengue outbreaks [54]. This condition emphasizes the significance of educating the public about proper management tools and strategies for managing and mitigating the spread of public health pests, including bed bugs and mosquitoes.

Even though the DIY method is generally preferred by the public, it can be hazardous to one’s health [39]. Some frequent DIY ways for eradicating bed bugs include utilizing untested insecticides or disposing of contaminated furniture without first treating it [49]. However, misuse of pesticides, especially those that are not labeled for bed bugs, might result in control failure. Intoxication can also result from failing to use products as directed on their labels [39]. The Center for Disease Control and Prevention (CDC), United States, has reported an alarmingly high number of 111 cases of sickness related to misuse of insecticides, such as pyrethroids and pyrethrins to control bed bugs [55]. In addition, the usage of over-the-counter products for home treatment may lead to the increase in bed bugs resistance. A study conducted in the United States discovered that the long-term use of pyrethroid insecticides causes an increase in the *kdr* mutations in the *C*. *lectularius* population, which plays a role in insecticide resistance [56]. Similarly, a high prevalence of pyrethroid resistance associated with *kdr* mutations was also observed in the tropical bed bug, *C*. *hemipterus* [57,58]. As a result, public education about bed bug’s biology, clinical repercussions, and effective eradication methods is vital for bed bug prevention and management.

In conclusion, it is imperative that PCOs and researchers work together to educate the public, particularly to reduce misunderstandings regarding bed bugs and effective bed bug control measures. Future research should involve participants from the hospitality industry, medical professionals, and pest control companies to fully understand the effects of bed bug infestations in Indonesia.

## Supporting information

S1 Checklist STROBE statement—checklist of items that should be included in reports of observational studies. (DOCX)

S1 Dataset Perception on Bed Bugs Infestation in Indonesia (Survey Tingkat Kesadaran Masyarakat Terhadap Serangan Kutu Busuk Di Indonesia). This dataset is also available at (https://bit.ly/3aLldce).
(PDF)
