# Supplementary material for: Perception, attitudes, and knowledge on infestation and management of bed bugs in major cities of Indonesia: A cross-sectional online survey
Source: PLoS One. 2023 Jul 27;18(7):e0288682. doi: 10.1371/journal.pone.0288682 (PMC10374038; doi:10.1371/journal.pone.0288682)
Supplement: S1 Checklist — (DOCX) [file pone.0288682.s001.docx]

STROBE Statement—checklist of items that should be included in reports of observational studies

|  | Item No. | Recommendation | Page  No. | Relevant text from manuscript |
| --- | --- | --- | --- | --- |
| **Title and abstract** | 1 | (*a*) Indicate the study’s design with a commonly used term in the title or the abstract | 1 (Line 11) | Perception, attitudes, and knowledge on infestation and management of bed bugs in Indonesia: a cross-sectional online survey |
|  |  | (*b*) Provide in the abstract an informative and balanced summary of what was done and what was found | 2 (Lines 29-37) | Herein, this study investigates the public’s perception, attitudes, and knowledge on bed bug-related issues in Indonesia through an online survey. Despite low case reports, three in five Indonesians (600 respondents) have encountered bed bugs at least once; mostly in their homes (74.1%). Approximately half of the respondents correctly identified bed bugs, whereas mites were often misidentified for bed bug (26.3%). |
| Introduction | | | |  |
| Background/rationale | 2 | Explain the scientific background and rationale for the investigation being reported | 4 (Lines 87 – 95) | The lack of reporting on bed bugs in Indonesia may give a false impression on the actual infestation levels of this pest in the country. |
| Objectives | 3 | State specific objectives, including any prespecified hypotheses | 5 (Lines 95 – 97) | The purpose of this study is to assess the public's awareness, attitudes, and perception of the infestation and management of bed bugs in Indonesia, based on online questionnaires. |
| Methods | | | |  |
| Study design | 4 | Present key elements of study design early in the paper | 6 (Lines 122 – 136) | The questionnaire was divided into five main sections. |
| Setting | 5 | Describe the setting, locations, and relevant dates, including periods of recruitment, exposure, follow-up, and data collection | 6 (Lines 112 – 116) | The study took place from May to October 2021. The questionnaire was presented in both Bahasa Indonesia and English. |
| Participants | 6 | (*a*) *Cohort study*—Give the eligibility criteria, and the sources and methods of selection of participants. Describe methods of follow-up  *Case-control study*—Give the eligibility criteria, and the sources and methods of case ascertainment and control selection. Give the rationale for the choice of cases and controls  *Cross-sectional study*—Give the eligibility criteria, and the sources and methods of selection of participants | 6 (Lines 116 – 118) | The link for the online survey was shared with the public through various social networking platforms with public access, such as university and research-related Facebook groups, Instagram, and Twitter accounts. |
|  |  | (*b*) *Cohort study*—For matched studies, give matching criteria and number of exposed and unexposed  *Case-control study*—For matched studies, give matching criteria and the number of controls per case |  |  |
| Variables | 7 | Clearly define all outcomes, exposures, predictors, potential confounders, and effect modifiers. Give diagnostic criteria, if applicable | 6-7 (Lines 101 – 151) | Respondents who answered "Yes" to the statement "I have seen a bed bug" but selected the incorrect image of the insect were considered to have misidentified the bed bug. |
| Data sources/ measurement | 8* | For each variable of interest, give sources of data and details of methods of assessment (measurement). Describe comparability of assessment methods if there is more than one group | 7 (Lines 141 – 153) | Respondents’ demographic characteristics and questions with yes or no answers were analyzed as percentages. Meanwhile, Likert scale responses were presented as frequencies. |
| Bias | 9 | Describe any efforts to address potential sources of bias | 5 (Lines 104 – 106) | The results of this study therefore reflect the views and knowledge of Indonesian online users, most likely those from urban areas, and may not necessarily reflect the country's entire population. |
| Study size | 10 | Explain how the study size was arrived at |  |  |

Continued on next page

| Quantitative variables | 11 | Explain how quantitative variables were handled in the analyses. If applicable, describe which groupings were chosen and why | 7 (Lines 143 – 151) | The Kruskal-Wallis H test was used to analyze the influence of different age groups on their experience in encountering bed bug infestations. . For Likert scale responses, we divided the respondents into two groups based on three categories: i) education background (those with less than a bachelor's degree and the remaining respondents), ii) gender (male and female respondents), and iii) age (respondents below the age of 30 and 30 years old and above). |
| --- | --- | --- | --- | --- |
| Statistical methods | 12 | (*a*) Describe all statistical methods, including those used to control for confounding | 7 (Lines 143 – 145, 150 – 152) | The Kruskal-Wallis H test was used to analyze the influence of different age groups on their experience in encountering bed bug infestations. For binary questions (with yes-no answers), Fisher’s exact test was used to determine statistical significance between the two groups.  Descriptive statistics and Mann-Whitney U test were used to test for any significant differences between these respective groups in Likert scale responses. |
|  |  | (*b*) Describe any methods used to examine subgroups and interactions | 7 (Line 142-143) | For binary questions (with yes-no answers), Fisher’s exact test was used to determine statistical significance between the two groups. |
|  |  | (*c*) Explain how missing data were addressed |  |  |
|  |  | (*d*) *Cohort study*—If applicable, explain how loss to follow-up was addressed  *Case-control study*—If applicable, explain how matching of cases and controls was addressed  *Cross-sectional study*—If applicable, describe analytical methods taking account of sampling strategy |  |  |
|  |  | (*e*) Describe any sensitivity analyses |  |  |
| Results | | | | |
| Participants | 13* | (a) Report numbers of individuals at each stage of study—eg numbers potentially eligible, examined for eligibility, confirmed eligible, included in the study, completing follow-up, and analysed | 15 (Lines 159, 166) | The online survey received 600 responses, with nearly equal numbers of female (56.5%) and male (43.5%) respondents. 362 of the 600 respondents indicated they have encountered bed bugs. |
|  |  | (b) Give reasons for non-participation at each stage |  |  |
|  |  | © Consider use of a flow diagram |  |  |
| Descriptive data | 14* | (a) Give characteristics of study participants (eg demographic, clinical, social) and information on exposures and potential confounders | 15 (159 – 166) | Most of respondents (35.8%) were between the ages of 31 and 40, followed by those between the ages of 41 and 50 (19.3%). |
|  |  | (b) Indicate number of participants with missing data for each variable of interest |  |  |
|  |  | © *Cohort study*—Summarise follow-up time (eg, average and total amount) |  |  |
| Outcome data | 15* | *Cohort study*—Report numbers of outcome events or summary measures over time |  |  |
|  |  | *Case-control study—*Report numbers in each exposure category, or summary measures of exposure |  |  |
|  |  | *Cross-sectional study—*Report numbers of outcome events or summary measures | Table 1 |  |
| Main results | 16 | (*a*) Give unadjusted estimates and, if applicable, confounder-adjusted estimates and their precision (eg, 95% confidence interval). Make clear which confounders were adjusted for and why they were included | 17 (Lines 194 – 199,  229 – 235) | The ability to distinguish bed bugs does not differ significantly between respondents aged 30 and older (n= 273) and those aged under 30 (n= 89) (Fisher's exact test, p = 0.110).  Male respondents displayed a higher positive attitude than female respondents in distinguishing bed bug bites from other insects (Mann-Whitney U = 38593.50, p = 0.006) |
|  |  | (*b*) Report category boundaries when continuous variables were categorized |  |  |
|  |  | (*c*) If relevant, consider translating estimates of relative risk into absolute risk for a meaningful time period |  |  |

Continued on next page

| Other analyses | 17 | Report other analyses done—eg analyses of subgroups and interactions, and sensitivity analyses | 16 (Liens 187 – 192,  Table 2) | Among the 88 individuals older than 50, 81.0% reported having experienced bed bug infestations. This represents the greatest proportion of all age groups (Kruskal-Wallis H(5) = 27.74, p < 0.001). |
| --- | --- | --- | --- | --- |
| Discussion | | | | |
| Key results | 18 | Summarise key results with reference to study objectives | 23 (Lines 306 – 408) | In this study, the public’s awareness of bed bugs in Indonesia (73.0%) was found higher than in the United Kingdom (10.0%) (24) and Germany (13.0%) [25], but it was lower when compared with Ethiopia (91.6%) [26].  More than half of the respondents in this study claimed that they had been bitten by bed bugs or knew someone who had. Nonetheless, this data should be interpreted with caution as the respondents could easily mistake other insect bites for bed bug bite marks or vice versa. |
| Limitations | 19 | Discuss limitations of the study, taking into account sources of potential bias or imprecision. Discuss both direction and magnitude of any potential bias | 23 (Lines 306 – 312) | This survey was conducted online due to travel and movement limitations imposed by the Community Activities Restrictions Enforcement (CARE) in Indonesia at the time of the study due to the escalating COVID-19 outbreak. We recognize that our results could be impacted by the biases and constraints of the online platform. |
| Interpretation | 20 | Give a cautious overall interpretation of results considering objectives, limitations, multiplicity of analyses, results from similar studies, and other relevant evidence | 24 (Lines 313 – 342) | In this study, the public’s awareness of bed bugs in Indonesia (73.0%) was found higher than in the United Kingdom (10.0%) (24) and Germany (13.0%) [25], but it was lower when compared with Ethiopia (91.6%) [26]. |
| Generalisability | 21 | Discuss the generalisability (external validity) of the study results | 24-25 (Lines 327 – 342) | A review by Schoelitsz et al. (2019) [38] described that the ability to accurately identify household insect pests depends on a prior encounter or negative experience with the pests. Thus, the low level of bed bug awareness in Europe (the United Kingdom and Germany) compared with Indonesia and Ethiopia could be ascribed to a lower level of bed bug infestation rate in that region. Furthermore, the bed bug infestation may have been kept under control in Europe due to the availability and application of the code of practice and guidelines for bed bug management. |
| Other information | |  | | |
| Funding | 22 | Give the source of funding and the role of the funders for the present study and, if applicable, for the original study on which the present article is based | 32 (Line 564) | This research was funded by Universiti Sains Malaysia, Research University Team (RUTeam) Grant Scheme (Grant number: 1001/PCCB/8580041) to GVS. We also thank the Fulbright Malaysian Scholar Program for supporting research attachment of GVS at the University of California Riverside. The funders had no role in study design, data collection and analysis, decision to publish, or preparation of the manuscript. |

*Give information separately for cases and controls in case-control studies and, if applicable, for exposed and unexposed groups in cohort and cross-sectional studies.

**Note:** An Explanation and Elaboration article discusses each checklist item and gives methodological background and published examples of transparent reporting. The STROBE checklist is best used in conjunction with this article (freely available on the Web sites of PLoS Medicine at http://www.plosmedicine.org/, Annals of Internal Medicine at http://www.annals.org/, and Epidemiology at http://www.epidem.com/). Information on the STROBE Initiative is available at www.strobe-statement.org.
