# Supplementary material for: Perception, attitudes, and knowledge on infestation and management of bed bugs in major cities of Indonesia: A cross-sectional online survey
Source: PLoS One. 2023 Jul 27;18(7):e0288682. doi: 10.1371/journal.pone.0288682 (PMC10374038; doi:10.1371/journal.pone.0288682)
Supplement: S1 Dataset — This dataset is also available at (https://bit.ly/3aLldce). (PDF) [file pone.0288682.s002.pdf]

S1 Dataset: Survey Tingkat Kesadaran Masyarakat Terhadap Serangan Kutu Busuk Di Indonesia/Survey of People's Perceptionon Bed Bugs Infestation in Indonesia

Penelitian survey ini bertujuan untuk mengetahui tingkat kesadaran masyarakat Indonesia terhadap gangguan kutu busuk/kutu kasur/bangsat/kepinding/tinggi/tumila di rumah atau tempat umum lainnya. Selain itu, dari survey ini kami juga ingin melihat preferensi masyarakat terhadap jasa pengendalian kutu busuk yang ditawarkan oleh perusahaan swasta. Waktu pengisian kuisioner ini kurang lebih 3 - 5 menit. Kuesioner ini disajikan dalam dua bahasa, yaitu bahasa Indonesia dan bahasa Inggris. Jika terdapat perbedaan arti dalam dua bahasa tersebut, maka versi bahasa Indonesia dapat digunakan sebagai rujukan yang benar.
Dibawah ini terdapat sejumlah pertanyaan tentang pengalaman mengenai serangan kutu busuk dan juga pengendaliannya di masyarakat Indonesia. Mohon kesediaan responden untuk menjawab sesuai kondisi yang pernah dialami. Bentuk pertanyaan terdiri dari; pertanyaan dengan jawaban ya atau tidak, pilihan gambar, isian, dan skala 1 -5. Untuk pertanyaan dengan jawaban skala 1-5, isilah dengan keterangan skala dibawah ini :
1: Sangat Tidak Setuju2:
Tidak Setuju
3: Netral
4: Setuju
5: Sangat Setuju

The objective of this survey is to learn public awareness on bedbugs infestation at home or other public places. Moreover, from this survey we want to see people's preferences for bed bug control services offered by private companies. The time to fill out this questionnaire is approximately 3-5 minutes.

The questionnaire was provided in two languages; Bahasa Indonesia and English. Wherever there is a discrepancy, the original meaning in Bahasa Indonesia is used as correct version.
This questionnaire contains number of questions regarding public experience on bedbugs attack and how to control it. Please answer the questions according to respondent's own experience. Types of question is including question with yes or no answer, image options answer, short answer, and 1-5 scale answer. For question with 1-5 scale answer please fill it according to the information below:
1: Strongly Disagree
2: Disagree
3: Neutral
4: Agreed
5: Strongly Agree

* Indicates required question

Profil Responden / Respondent's Profile

1. Jenis Kelamin / Gender *

Mark only one oval.

- ( ) Perempuan / Female
- ( ) Laki-laki / Male

2. Domisili (kota/kabupaten) / Residency (city) *

____

3. Umur / Age *

Mark only one oval.

- ( ) < 15
- ( ) 15 - 20
- ( ) 21 - 25
- ( ) 26 - 30
- ( ) 31 - 40
- ( ) 41 - 50
- ( ) > 50

4. Tingkat Pendidikan / Level of Education *

Mark only one oval.

- SD / Primary School
- SMP / Junior High School
- SMA / High School
- D3 / Diploma
- S1 / Bachelor Degree
- S2 / Master Degree
- S3 / Doctoral Degree

Pengalaman dan Pengetahuan Tentang Kutu Busuk / Experience and Knowledge on Bed Bug

5. Saya pernah melihat kutu busuk secara langsung (Jika iya, lanjut ke pertanyaan nomor 2. Jika tidak lanjut ke pertanyaan nomor 5) / I have seen (encountered) bed bugs. (If Yes, go to question number 2. If No, continue to question number 5) *

Mark only one oval.

- Ya / Yes
- Tidak / No

6. Kapan anda melihat kutu busuk secara langsung (bulan/tahun)? / When did you see (encountered) the bed bugs?

Mark only one oval.

- 2021 - sekarang / now
- 2011 - 2020
- 2001 - 2010
- 1991 - 2000
- < 1990

7. Dimana anda melihat kutu busuk secara langsung? / Where did you see (encountered) the bed bugs?

Check all that apply.

- [ ] Rumah / Home
- [ ] Penginapan / Hotel
- [ ] Bioskop / Cinema
- [ ] Transport Publik / Public Transport
- [ ] Other: ____________________

8. Sudah berapa kali anda melihat kutu busuk secara langsung? / How many times did you see (encountered) the bed bugs?

Mark only one oval.

- ( ) 1 - 2
- ( ) 3 - 5
- ( ) > 5

9. Saya mengetahui bentuk kutu busuk / I know the shape or form of bed bugs *

Mark only one oval.

- ( ) Ya/Yes
- ( ) Tidak/No

10. Manakah dari 5 gambar serangga disamping yang merupakan kutu busuk? / Which one is the bed bug from the 5 pictures below? *

Mark only one oval.

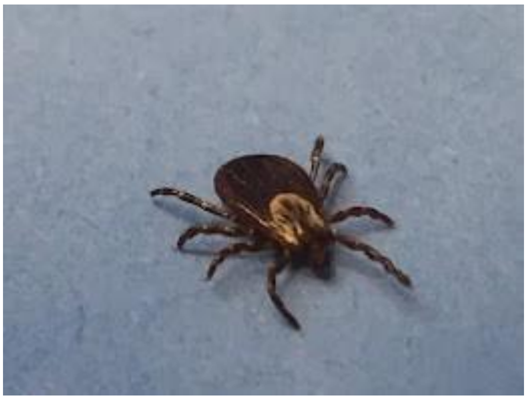

Gambar 1 / Picture 1

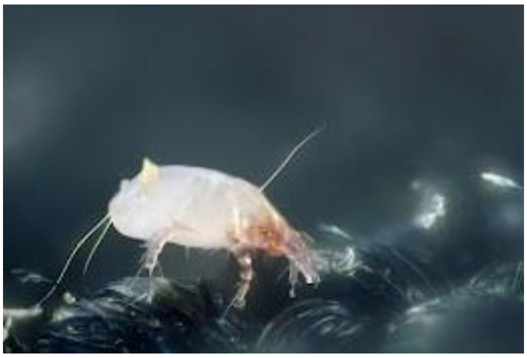

Gambar 2 / Picture 2

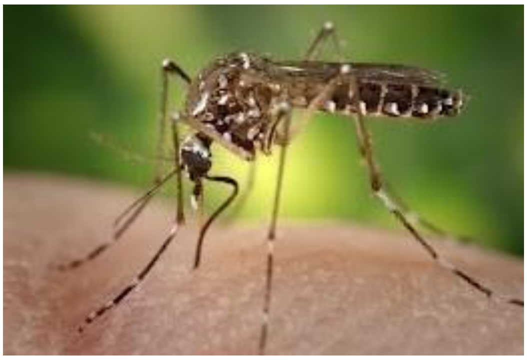

Gambar 3 / Picture 3

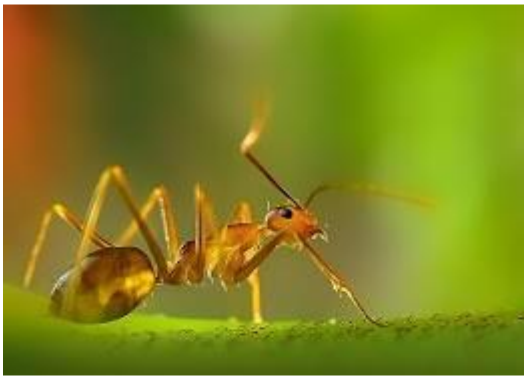

Gambar 4 / Picture 4

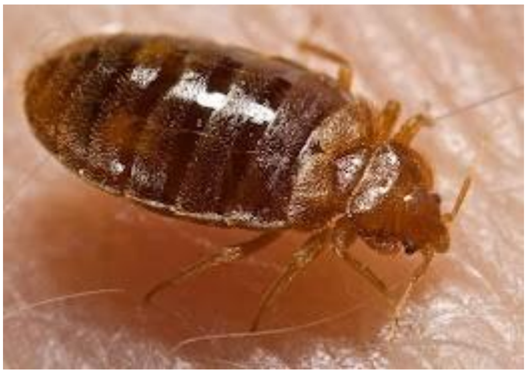

Gambar 5 / Picture 5

Gigitan Kutu Busuk / Bed Bug's Bite

11. Saya dapat membedakan gigitan kutu busuk dengan gigitan serangga lainnya / I can differentiate bed bug's bite apart from those of other insects *

Mark only one oval.

Sangat Tidak Setuju/Strongly Disagree

1 ◯

2 ◯

3 ◯

4 ◯

5 ◯

Sangat Setuju/Strongly Agree

12. Saya mengetahui kalau gigitan kutu busuk dapat mengakibatkan gatal dan munculnya ruam kemerahan di tempat gigitan / I know that bed bug's bite can cause itchy and rash *

Mark only one oval.

Sangat Tidak Setuju/Strongly Disagree

1	◯
2	◯
3	◯
4	◯
5	◯

Sangat Setuju/Strongly Agree

13. Saya pernah atau mengetahui seseorang yang pernah digigit kutu busuk dan mengalami reaksi gatal dan ruam / I have been or knew someone who has been bitten by bed bugs and had an allergic reaction *

Mark only one oval.

◯ Ya / Yes
◯ Tidak / No

14. Jika muncul ruam kemerahan di kulit, saya akan menganggap itu sebagai tanda gigitan kutu busuk / If a reddish rash appears on the skin, I will assume it as a bed bug's bite *

Mark only one oval.

Sangat Tidak Setuju/Strongly Disagree

1 ⬭
2 ⬭
3 ⬭
4 ⬭
5 ⬭

Sangat Setuju/Strongly Agree

15. Saya pernah atau mengetahui seseorang yang digigit kutu busuk di tempat umum / I have been or knew someone who has been bitten by bed bugs in a public places *

Mark only one oval.

⬭ Ya / Yes
⬭ Tidak / No

Infestasi Kutu Busuk di Tempat Umum / Bed Bug Infestation in Public Places

16. Tidak hanya di rumah, kutu busuk juga dapat menyerang tempat umum seperti hotel, bioskop, dan transportasi umum / Apart from home, bed bugs can also infest public places such as hotel, cinemas, and public transportation *

Mark only one oval.

Sangat Tidak Setuju/Strongly Disagree

1

2

3

4

5

Sangat Setuju/Strongly Agree

17. Setiap saya pergi ke tempat umum (hotel, kantor, transportasi umum) saya akan mengecek apakah ada kutu busuk ditempat tersebut / Every time I go to a public place (e.g., hotel, office, public transportation) I will check for bed bugs *

Mark only one oval.

Sangat Tidak Setuju/Strongly Disagree

1 ⬭

2 ⬭

3 ⬭

4 ⬭

5 ⬭

Sangat Setuju/Strongly Agree

18. Saya menganggap bahwa serangan kutu busuk di rumah dan tempat umum menganggu / I find bed bug infestation at home and in public places as annoying *

Mark only one oval.

Sangat Tidak Setuju/Strongly Disagree

1 ⬭

2 ⬭

3 ⬭

4 ⬭

5 ⬭

Sangat Setuju/Strongly Agree

Pengendalian Serangan Kutu Busuk / Bed bugs Control and Management

19. Saya pernah mencari informasi mengenai cara – cara mengendalikan serangan kutu busuk / I have looked for information on ways to control bed bug infestation *

Mark only one oval.

⬭ Ya / Yes

⬭ Tidak / No

20. Saya mengetahui adanya insektisida khusus untuk membasmi kutu busuk yang dijual secara bebas di pasaran / I know that there are insecticide specific for bed bugs available *

Mark only one oval.

- Ya / Yes
- Tidak / No

21. Saya mengetahui ada perusahaan yang menawarkan jasa pengendalian hama kutu busuk / I know there are local companies that offers bed bug control services *

Mark only one oval.

- Ya / Yes
- Tidak / No

22. Saya pernah meminta bantuan jasa pengendalian hama saat rumah atau tempat lain diserang hama kutu busuk / I have asked the services from pest control operator for inspection and controlling bedbugs at my house *

Mark only one oval.

- Ya / Yes
- Tidak / No

23. Saat terjadi serangan kutu busuk di rumah saya, saya akan meminta bantuan pest control atau jasa pengendalian dari perusahaan pengendalian hama / When there is a bed bug infestation in my house, I will ask pest control operator for help *

Mark only one oval.

Sangat Tidak Setuju/Strongly Disagree

1 ⬭

2 ⬭

3 ⬭

4 ⬭

5 ⬭

Sangat Setuju/Strongly Agree

24. Jika rumah saya terserang hama kutu busuk saya akan memilih membasminya secara mandiri dibandingkan dengan meminta jasa professional agar lebih hemat / I would rather do it by myself to control bedbug infestation at my house instead of Pest Control Operators because it is more economical *

Mark only one oval.

Sangat Tidak Setuju/Strongly Disagree

1 ⬭

2 ⬭

3 ⬭

4 ⬭

5 ⬭

Sangat Setuju/Strongly Agree

25. Saya akan mencari informasi lebih dalam mengenai perusahaan yang menawarkan jasa pengendalian hama kutu busuk / I'm going to find out more about a companies that offers bed bug control services *

Mark only one oval.

Sangat Tidak Setuju/Strongly Disagree

1 ◯

2 ◯

3 ◯

4 ◯

5 ◯

Sangat Setuju/Strongly Agree

This content is neither created nor endorsed by Google.

Google Forms
